# Trends of sphincter-preserving surgeries for low lying rectal cancer: A 20-year experience in China

**DOI:** 10.3389/fonc.2022.996866

**Published:** 2022-12-08

**Authors:** Kuo Zheng, Qingqing Hu, Guanyu Yu, Leqi Zhou, Yuting Yao, Yuan Zhou, Hao Wang, Liqiang Hao, Enda Yu, Zheng Lou, Yongjing Zhang, Hong Qiu, Ronggui Meng, Wei Zhang

**Affiliations:** ^1^ Department of Colorectal Surgery, Changhai Hospital, Shanghai, China; ^2^ Global Epidemiology, Office of Chief Medical Officer, Johnson & Johnson, Shanghai, China; ^3^ Department of Professional Education, Johnson & Johnson Medical (Shanghai) LTD, Shanghai, China

**Keywords:** sphincter preservation, low rectal cancer, abdominoperineal resection, trends in surgery, low anterior resection, rectal cancer survival

## Abstract

**Background:**

Over the last 2 decades, patients with low rectal cancer have had better outcomes from improvements in surgical techniques in sphincter preservation. We aimed to quantify the trends in sphincter-preserving surgeries for low rectal cancer over 20 years in a top tertiary hospital in China.

**Methods:**

Between 1999 and 2021, a cohort of patients with primary malignant rectal tumor ≤5cm from the anal verge and who received elective surgeries at Changhai Hospital, Shanghai, China, was identified. Data were extracted from electronic medical records. A Joinpoint Regression Model was used to analyze trends in surgical procedures by average annual percentage change (AAPC). Adjusted Cox proportional hazards regression model was used to assess overall survival.

**Results:**

Among a total of 4,172 patients during the study period, 3,111 (74.6%) underwent a sphincter-preserving surgery and 1,061 (25.4%) received APR. Sphincter-preserving surgery increased 3.6% per year (95%CI, 2.3-4.9). Low anterior resection was the most performed procedure (86.3%) and maintained a steady trend, while intersphincteric resection increased 49.4% annually (95%CI, 19.5-86.7) after initiation. Laparoscopic techniques increased 15.1% per year (95%CI, 8.4-43.4) after initiation. Sphincter-preserving surgery increased annually for tumors ≤2cm, 2-≤3cm and 3-≤4cm from the anal verge (AAPC 7.1, 4.5-9.8; 4.7, 3.1-6.3; 2.7, 1.7-3.6, respectively). Furthermore, patients with sphincter-preserving surgery had a better overall survival than abdominoperineal resection (APR) patients (adjusted HR 0.78, 95% CI, 0.65-0.93, p=.01).

**Conclusions:**

Utilization of sphincter-preserving surgeries increased significantly over the last 20 years. Patients with low rectal cancer who underwent sphincter preservation had better survival than similar patients who underwent APR.

## Introduction

China is undergoing an increasing burden of rectal cancer (1). Data from Chinese National Central Cancer Registry reported a 2% annual increase in the age-standardized incidence of rectal cancer in rural areas from 2005 to 2015 (2, 3). GLOBOCAN estimated that new cases and deaths from rectal cancer in China from 2020 to 2040 would increase 55.4% and 81.0%, respectively (4).

Low rectal cancer is generally considered as ≤5cm from the lower edge of the tumor to the anal verge (5, 6). Surgery is one of the major curative treatments available (7). However, there is an absence of standardization of surgical procedures and approach in the low rectum, partly because of a difficulty in maneuvering in the narrow space in the rectum as well as the rapid development of surgical techniques (8).

Patients with low rectal cancer have traditionally been treated surgically with an abdominoperineal resection (APR) (9) Over the last 2 decades, patients with low rectal cancer have had better function and quality of life (10) from improvements in surgical techniques in sphincter preservation, such as anterior resection (AR) by Dixon in 1948 (11), and intersphincter resection (ISR) by Schiessel et al. in 1994 (12). However, sphincter-preserving surgery is more challenging to operate than APR due to the anatomic features of the deep and narrow pelvic cavity, and thus is highly dependent on the experience of the surgeon (13). Moreover, some patients with ultra-low tumors ≤2cm from the anal verge will still require an APR due to the closeness of the tumor to the anal verge (7).

The laparoscopic technique has evolved rapidly since it was first introduced in the 1980s in colorectal surgery (14). It has been widely accepted in the surgical community for colorectal surgery, with advantages in perioperative morbidity and mortality (14, 15). However, laparoscopic surgery is technically demanding due to restricted movement of the rigid instruments in a narrow deep pelvic cavity and may lead to a longer operation time compared to open surgery for low rectal cancer (16).

Although previous studies have investigated the short and long-term oncological outcomes of novel surgeries (17–25), the studies for trends in sphincter-preserving surgeries usage for low rectal cancer are still lacking. An analysis of trends in sphincter-preserving surgeries will provide real-world evidence for the applications of surgical techniques that may benefit patients’ outcomes and life. Therefore, we conducted a study summarizing past practices in low rectal surgery over 20 years at one top tertiary hospital in China. The objective of our study was to quantify the trends in procedures and approaches of sphincter-preserving surgeries for low rectal cancer. Furthermore, we evaluated overall survival for patients who had undergone sphincter-preserving surgery compared with APR.

## Methods

### Study population

An initial cohort of rectal cancer patients was identified at Changhai Hospital between November 1999 and October 2021. Patients eligible for study were diagnosed with an invasive and resectable first primary rectal cancer and underwent an elective surgery at the hospital. A resectable tumor was evaluated by the surgeons and multi-disciplinary team that the tumor could be resected by the curative-intent surgery. Patients were excluded from the study if they had a tumor distance >5cm from the lower edge of the tumor to the anal verge, were younger than 18 years, or were diagnosed with multiple tumors. Patients with unknown surgical procedures were also excluded (**Supplemental Figure 1**).

### Data sources

This is a non-randomized, non-interventional study of electronic medical record (EMR) from the Department of Colorectal Surgery, Changhai Hospital at Shanghai. Study data were from the EMR database initiated in the department in November 1999, which included colorectal cancer patients who had undergone elective surgeries consecutively at the Department and signed the consent forms. Each patient was assigned a unique code for a standardized treatment care and follow-up. The elective surgery was performed according to yearly guidelines of National Comprehensive Cancer Network (NCCN) and Chinese Society and Clinical Oncology (CSCO). The database included details on demographic and clinicopathological characteristics; surgery related information including the date of surgery, procedure, laparoscopic or open surgery; and follow-up information for the date of follow-up, vital status and the date of death, if applicable. Follow-up data were collected from outpatient EMR records at the hospital; if the records were not available, a phone call interview was conducted to follow up with the patient or relatives if the patient had died. Three attempts to contact non-responders by phone were made. Patients were followed up at 3-month intervals for 2 years, then at 6-month intervals for the next 3 years, and once annually thereafter. Patients were deemed “non-follow-up” if they did not have any follow-up information. Characteristics for the non-follow-up group and follow-up group were assessed. Among patients who had at least one follow-up information, patients were deemed “lost to follow-up” if they could not be contacted through both outpatient EMR and phone call interviews on the dates of follow-up. The reasons of loss-to-follow-up by phone call were recorded, such as follow-up refusal, wrong number or non-existing number, etc.

All the data for the study were de-identified. The study was approved by the Institutional Review Board at the hospital (IRB number: CHEC2022-021).

### Study outcomes

The main outcomes were the trends in sphincter-preserving surgical procedures and approach for patients with low rectal cancer. Trends were expressed by the average annual percentage change (AAPC), which was a weighted summary measure taking into account the trend transitions (joinpoints) to describe the AAPC over a period of multiple years (26). For more than one joinpoint or two segments, the annual percentage change (APC) was described for each segment. When describing the trends with APCs or AAPCs, “increase” or “decrease” were used if slope of the trend was statistically significant (p<0.05); otherwise, “stable” or “non-significant increase” or “non-significant decrease” were used.

Overall survival was evaluated for patients who were not deemed as non-follow-up, as defined above. Those with follow-up and non-follow-up were evaluated for baseline characteristics to assess the presence of selection bias. Overall survival was defined as the period between the date of surgery and death of any cause or last follow-up, whichever came first. Patients were censored at the time of their last follow-up if they were alive or lost-to-follow-up.

### Statistical analysis

The demographic and clinicopathological categorical characteristics of patients who had undergone sphincter-preserving surgery and APR were described by frequencies and percentages. Categorical variables were compared with the chi-square or Fisher’s exact test, and continuous variables will be compared with the analysis of variance (ANOVA) test, between the two groups. Missing data were described by reporting the proportion of missing data for that variable and the missing values were not imputed.

The National Cancer Institute’s Joinpoint Regression Analysis Program (version 4.9.0.0) (26) was used to calculate the APCs and AAPCs and corresponding 95% confidence intervals (CIs) to quantify the trends in direction and magnitude in surgical procedures and approach, for patients with low rectal cancer during the whole study period between 2000 and 2021, and the last five years between 2016 and 2021.

A Kaplan-Meier curve and adjusted Cox proportional hazards regression model were used to assess the overall survival for patients with sphincter-preserving surgery compared with APR during the whole study period in 2000-2021, as well as three fixed intervals in 2000-2008, 2009-2015, and 2016-2021, respectively. The model was adjusted for potential confounders, which were assessed *a priori* based on the three confounder properties (27, 28), and included demographic (year of surgery groups for 2000-2021, age groups, gender, tumor location groups, tumor size) and clinical, pathological and treatment variables (baseline comorbidities, neoadjuvant therapy, pathological stage, histology, grading). The reference group for each confounder was listed as follows, year of surgery: 2000-2008, age: 46-65 years, gender: male, tumor location: 4-≤5cm, baseline comorbidities: no; neoadjuvant therapy: no; pathological stage: stage 0 and stage I; histology: common adenocarcinoma; grading: G1 well differentiation. Tumor location was measured through digital rectal examination, colonoscopy or rigid sigmoidoscope/proctoscopy in clinical examination before surgery. The proportional hazards assumption was assessed using the Schoenfeld and scaled Schoenfeld residuals. The proportionality was satisfied if p>0.05 (29).

### Subgroup and sensitivity analysis

Subgroup analysis of trend was performed for age-specific (18-45, 46-65, and 66-97) and sex-specific (male and female) groups. Sensitivity analysis of survival was performed for patients followed-up in one year, three years and five years, to evaluate the consistency of the survival results.

All statistical analyses were conducted using RStudio (2020) (30). All P values were 2-sided, and point estimates were presented with 95% CIs. The significance level was set at P ≤ 0.05 for all analyses.

## Results

### Patient characteristics

In our cohort, a total of 4,172 patients had low rectal tumor and received elective surgeries at the study site during the study period (**Supplemental Figure 1**). The characteristics between those follow-up and non-follow-up patients were comparable (**Supplemental Table 1**). “Non-follow-up” patients were not included in the survival analysis. The mean (SD) age of patients at surgery was 59.1 (12.0) years; more than a half of patients (54.3%) were 45-65 years old. Most of the patients were males (61.9%) (**Table 1**). When categorized by sphincter-preserving surgery and APR, ultra-low tumors (≤2cm) accounted for 8.4% of sphincter-preserving surgery and 43.2% of APR (p<.0001). A pathological advanced stage (II and III) was accounted for 56.4% of sphincter-preserving surgery and 61.7% of APR (p=0.014). Moreover, APR group was more likely to have poorly differentiated grading compared to sphincter-preserving group (p=0.0004). Neoadjuvant therapy was employed among 20.2% of APR group, higher than 17.0% of sphincter-preserving surgery group (p<.0001).

**Table 1:** Characteristics of patients with low rectal cancer, stratifying by sphincter-preserving surgery and APR.

Characteristics	Overall	Sphincter-preserving surgery	APR	P value^5^
	(N=4,172)	(N=3,111, 74.6%)	(N=1,061, 25.4%)	
Year of surgery				<.0001
2000-2008	837 (20.1)	477 (15.3)	360 (33.9)	
2009-2015	1,403 (33.6)	986 (31.7)	417 (39.3)	
2016-2021	1,932 (46.3)	1,648 (53.0)	284 (26.8)	
Age, years, mean (SD)	59.1 (12.0)	59.2 (12.0)	59.0 (12.0)	0.85
Age group, years				0.97
18-45	584 (14.0)	438 (14.1)	146 (13.8)	
46-65	2264 (54.3)	1687 (54.2)	577 (54.4)	
66-97	1324 (31.7)	986 (31.7)	338 (31.9)	
Gender				0.47
Female	1589 (38.1)	1175 (37.8)	414 (39.0)	
Male	2583 (61.9)	1936 (62.2)	647 (61.0)	
Baseline comorbidities	2110 (50.6)	1600 (51.4)	510 (48.1)	0.058
Neoadjuvant therapy	742 (17.8)	528 (17.0)	214 (20.2)	0.018
Tumor location, cm, median (IQR) ^1^	4.0 (3.0-5.0)	4.0 (3.0-5.0)	3.0 (2.0-3.5)	<.0001
Tumor location group, cm				<.0001
≤2cm	731 (17.5)	270 (8.7)	459 (43.3)	
2-≤3cm	1033 (24.8)	701 (22.5)	332 (31.3)	
3-≤4cm	1155 (27.7)	968 (31.1)	187 (17.6)	
4-≤5cm	1253 (30.0)	1172 (37.7)	81 (7.6)	
Tumor size, cm, median (IQR)	3.5 (2.5-4.5)	3.5 (2.5-5.0)	3.5 (3.0-4.5)	0.97
Surgery approach				<.0001
Open	3282 (78.7)	2361 (75.9)	921 (86.8)	
Laparoscopy	693 (16.6)	553 (17.8)	140 (13.2)	
Other ^2^	197 (4.7)	197 (6.3)	0 (0)	
Pathological stage				0.014
Stage 0	89 (2.1)	75 (2.4)	14 (1.3)	
Stage I	1205 (28.9)	926 (29.8)	279 (26.3)	
Stage II	1026 (24.6)	741 (23.8)	285 (26.9)	
Stage III	1383 (33.1)	1,014 (32.6)	369 (34.8)	
Stage IV	330 (7.8)	238 (7.6)	92 (8.7)	
Missing	139 (3.3)	117 (3.8)	22 (2.1)	
Tumor histology				0.16
Common type of adenocarcinoma	3297 (81.0)	2475 (81.8)	822 (78.5)	
Special type of adenocarcinoma	200 (10.9)	159 (10.2)	41 (15.2)	
Other ^3^	126 (6.9)	110 (7.0)	16 (5.9)	
Missing	4 (0.1)	4 (0.1)	0 (0)	
Histological grading ^4^				0.0004
Grade 1	72 (1.7)	50 (1.6)	22 (2.1)	
Grade 2	3217 (76.9)	2444 (78.3)	773 (72.7)	
Grade 3	404 (9.7)	272 (8.7)	132 (12.4)	
Grade 4	4 (0.1)	3 (0.1)	1 (0.1)	
Missing	486 (11.6)	351 (11.3)	135 (12.7)	
Total hospitalization days, median (IQR)	13 (10-19)	12 (9-16)	18 (13-24)	<.0001
Post-operative hospitalization days, median (IQR)	9 (6-12)	8 (6-10)	13 (9-17)	<.0001

APR, abdominoperineal resection.

^1^Tumor location was measured through colonoscopy or rigid sigmoidoscope/proctoscopy in clinical examination before surgery.

^2^Trans-anal resections.

^3^Included non-epithelial tumors (myogenic tumors, neurogenic tumors, GIST (gastrointestinal stromal tumors), lipomas and lipomatosis, tumor blood vessels, and other tumors).

^4^Grade 1 - well differentiated; Grade 2 - moderately differentiated; Grade 3 - Poorly Differentiated; Grade 4 – Undifferentiated.

^5^P values were calculated from Chi-square test or Fisher’s exact test for categorical variables and ANOVA test for continuous variables. Missing group was not incorporated into the test.

For surgical characteristics, a total of 3,111 (74.6%) patients with low rectal cancer underwent a sphincter-preserving surgery and the remaining 1,061 (25.4%) received APR (**Table 1**). The sphincter-preserving surgery was more likely to be performed in recent years 2016-2021 compared to APR (53.0% vs. 26.8%, p<.0001). Low anterior resection (LAR) was the most performed procedure of sphincter-preserving surgeries which accounted for 85.6%, while Intersphincter resection/Conformed sphincter-preserving operation (ISR/CSPO) was accounted for 5.7%.Approximately 16.6% of all patients underwent a laparoscopic surgery after the year of 2009, accounting for 17.8% of patients in the sphincter-preserving group and 13.2% in the APR group (p<.0001). The laparoscopic technique kept evolving and the proportion increased to 40%-50% patients in the last two years. Furthermore, APR group had a longer total hospitalization and post-operative hospitalization stay compared to sphincter-preserving group (p<.0001).

### Overall trend for sphincter-preserving surgery

**Figure 1:**
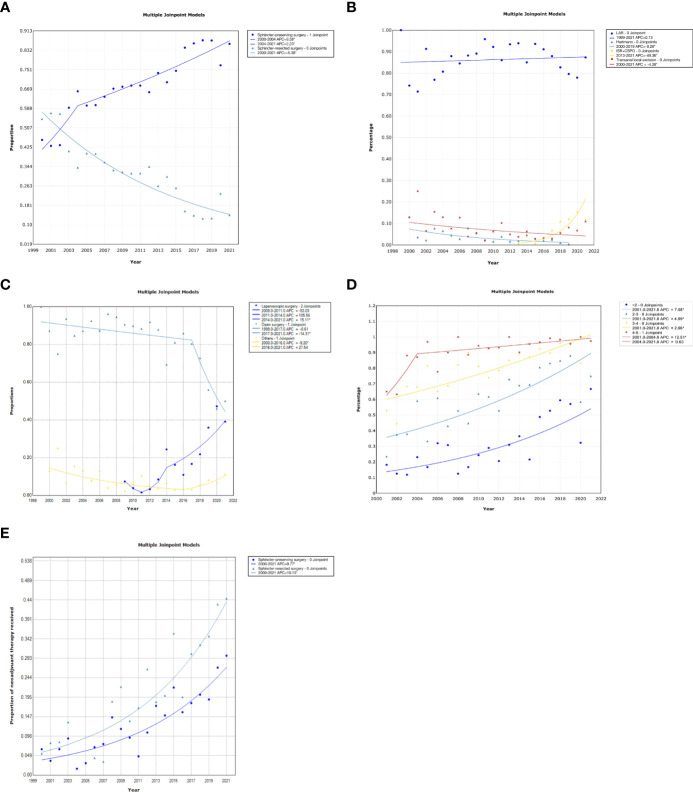
**(A-E)** Joinpoint regression models for overall trend in proportion of sphincter-preserving surgery **(A)**, sphincter-preserving procedures **(B)**, sphincter-preserving approach **(C)**, sphincter preservation for tumor location **(D)**, neoadjuvant therapy **(E)**.

Based on the Joinpoint regression model, the overall trend in proportion of sphincter-preserving surgery increased 3.6% per year (95%CI, 2.3-4.9) from 45.6% in 2000 to 85.9% in 2021 with a joinpoint of 66.0% in 2004 (**Table 2**; **Figure 1A**). The trends before and after 2004 were both increasing with an APC of 9.6 and 2.2, respectively (95% CI 2.7-16.9 for 2000-2004; 95% CI 1.5-3.0 for 2004-2021). The proportion of APR decreased 6.4% annually (95% CI, -7.7–5.0) from 54.4% in 2000 to 14.1% in 2021. When restricting to 2016-2021, the trends in sphincter-preserving and APR were at a stable increase or decrease.

**Table 2:** Trends in surgical techniques for low rectal cancer between 2000-2021 and 2016-2021.

	2000-2021		2016-2021	
Outcomes	Periods	APC (95% CI) (%)	AAPC (95% CI) (%)	AAPC (95% CI) (%)
Trend in proportions of surgical procedure				
Sphincter-preserving surgery	2000-2004	9.6^*^ (2.7-16.9)	3.6^*^ (2.3-4.9)	-0.7 (-4.1-2.8)
	2004-2021	2.2^*^ (1.5-3.0)		
LAR			0.1 (-0.4-0.7)	-1.7 (-5.5-2.1)
ISR/CSPO		49.4^*^ (19.5-86.7) ^1^	32.4^*^ (3.7-69.0)	
Hartmann	-9.3^*^ (-13.8–4.5)	-4.8 (-81.1-41.1)		
Transanal local excision	-1.5^*^ (-9.1-6.6)	29.1^*^ (12.2-48.4)		
APR	-6.4^*^ (-7.7–5.0)	2.8 (-12.6-21.1)		
Trend in proportions of surgical approach				
Laparoscopic surgery^2^	2009-2011	-53.0 (-81.8-21.2)	15.1^*^ (8.4-43.4) ^2^	33.1^*^ (15.8-52.9)
	2011-2014	105.6 (-20.3-430.2)		
	2014-2021	15.1^*^ (1.4-30.7)		
Open surgery	2000-2017	-0.6 (-1.5-0.3)	-3.3^*^ (-4.8–1.7)	-12.4^*^ (-17.6–6.8)
	2017-2021	-14.3^*^ (-21.4–6.6)		
Trend in percentages of sphincter-preserving surgery				
≤2cm		7.1^*^ (4.5-9.8)	0.1 (-17.0-20.8)	
2-≤3cm		4.7^*^ (3.1-6.3)	-3.9 (-12.7-5.9)	
3-≤4cm		2.7^*^ (1.7-3.6)	0.04 (-4.2-4.5)	
4-≤5cm	2000-2004	12.5^*^ (2.1-24.0)	2.3 (-0.9-3.8)	0.09 (-1.1-1.3)
	2004-2021	0.6 (-0.1-1.3)		

APC, annual percentage change; AAPC, average annual percentage change; 95% CI, 95% confidence interval; * The APC/AAPC was significantly different from zero (p < 0.05). The Joinpoint regression model was used to calculate the AAPC. If there were more than 1 joinpoint (2 line segments), APC was also calculated for each segment. 1. ISR/CSPO was initiated in 2013, therefore the overall trend period was 2013-2021 for ISR/CSPO. 2. Laparoscopic surgery was initiated in 2009, therefore the overall trend period was 2009-2021 for laparoscopic surgery.

For the surgical procedure, LAR was the most performed sphincter-preserving procedure and maintained a steady trend over the last 20 years. However, ISR/CSPO increased 49.4% annually from initiation of 0.5% in 2013 to 11.8% in 2021 (95%CI, 19.5-86.7), as well as increasing 32.4% annually (95%CI, 3.7-69.0) in 2016-2021 (**Table 2**; **Figure 1B**).

For the surgical approach, laparoscopic technique was first employed in 2009 and had a 15.1% annual increase in trend (95% CI, 8.4-43.4) to the absolute magnitude of 39.1% in 2021 (**Table 2**; **Figure 1C**). There were two joinpoints in 2011 and 2014, with a non-significant decrease and increase during 2009-2011 and 2011-2014 respectively, and a significant increase thereafter in 2014-2021 (APC 15.1, 95% CI, 1.4-30.7). When restricting to 2016-2021, laparoscopic surgery had a 33.1% annual increase (95%CI, 15.8-52.9). Correspondingly, the overall trend in the proportion of open surgery had a 3.3% annual decrease (95% CI, -4.8–1.7) over 20 years with a joinpoint in 2017. There was a non-significant decrease in 2000-2017 but a 14.3% annual decrease in 2017-2021.

Moreover, the trend in sphincter preservation for ultra-low and lower tumors (≤2cm, 2-≤3cm and 3-≤4cm) increased significantly annually and increased non-significantly for tumors at 4-≤5cm during 2000-2021 (AAPC, 95% CI: 7.1, 4.5-9.8; 4.7, 3.1-6.3; 2.7, 1.7-3.6; 2.3, -0.9-3.8, respectively) (**Table 2**; **Figure 1D**). However, there was an increasing trend in sphincter-preserving for tumors 4-≤5cm before 2004 (APC 12.5, 2.1-24.0). In 2016-2021, the trends in sphincter-preservation for different tumor locations were stable.

Neoadjuvant chemoradiotherapy was employed for minimizing the risk of locoregional recurrences but also for downsizing of the tumors near the anal sphincters to allow sphincter-preserving resection. In the last 20-years, the proportion of neoadjuvant therapy increased significantly among both sphincter-preserving and APR groups, with an annual increase of 9.7% and 10.1% respectively (**Figure 1E**).

The subgroup analysis on age-specific trends in sphincter-preserving surgeries increased 3.1% annually for patients aged 46-65 and 66-97 years but increased non-significantly for ages 18-45 years. The gender-specific trends in sphincter-preserving increased 4.6% annually for males but were stable for females over the 20 years (**Supplemental Table 2**).

### Overall survival

A total of 2,930 patients were included in the survival analysis. Patients had an average follow-up period of 35.0 months, with 32.6 months for the sphincter-preserving group and 44.6 months for the APR group. The sensitivity analyses for patients followed up for one, three and five years demonstrated consistent results with patients overall (**Supplemental Table 3**). The overall survival probability was 89.8% in the sphincter-preserving group and 74.7% in the APR group.

**Figure 2:**
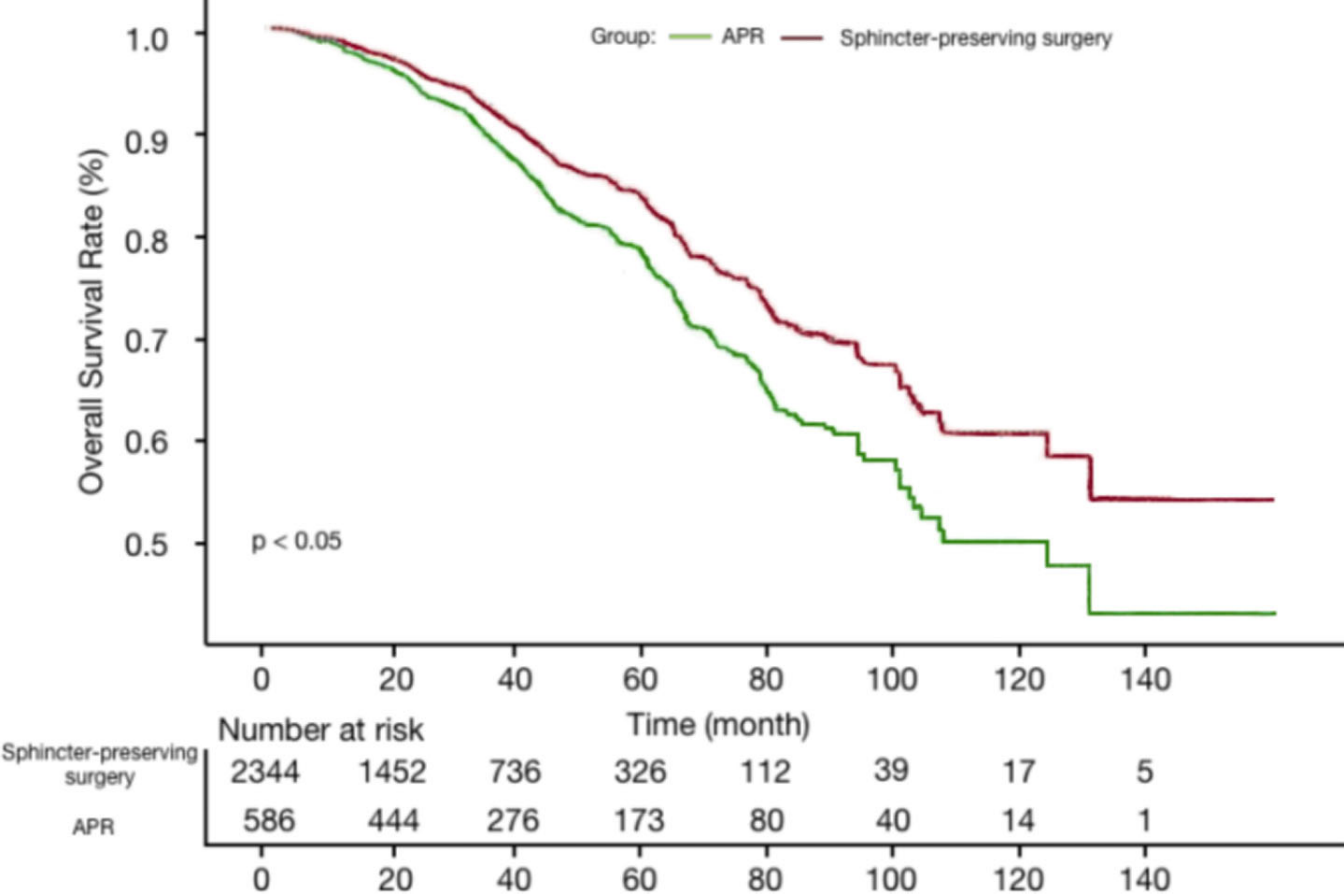
Kaplan-Meier curve for patients with sphincter-preserving surgery compared with APR. APR, Abdominoperineal resection.

The Kaplan-Meier curve showed that sphincter-preserving surgery was associated with better overall survival compared with APR (HR, 0.75; 95% CI, 0.63-0.90; log-rank P <0.05) (**Figure 2**). Moreover, our multivariable analysis based on the Cox regression model demonstrated similar findings, with significantly better survival for patients who had undergone sphincter-preserving surgery compared with APR (adjusted HR 0.78, 95% CI, 0.65-0.93, p=0.01) (**Table 3**). When stratifying for intervals in 2000-2008, 2009-2015, and 2016-2021, the results showed a better direction of overall survival for sphincter-preserving group than APR, although it did not reach statistical significance (**Table 3**).

**Table 3:** Hazard ratios (HRs) for overall mortality among patients undergone sphincter-preserving surgery compared with APR by year periods.

Sphincter-preserving surgery (vs. APR)	HR ^a^ (95% CI)	P
Overall		
2000-2021	0.78 (0.65-0.93)	0.01
Year at surgery		
2000-2008	0.76 (0.55-1.06)	0.42
2009-2015	0.81 (0.57-1.17)	0.26
2016-2021	0.75 (0.52-1.07)	0.11

APR, Abdominoperineal resection.

^a^Models were adjusted for demographic, clinical, pathological and treatment characteristics listed in **Table 1**, including year of surgery groups (for 2000-2021), age groups, gender, tumor location groups, tumor size, baseline comorbidities, neoadjuvant therapy, pathological stage, histology, grading.

## Discussion

Our study is the first to report the trends in sphincter-preserving surgeries in Chinese patients with low rectal cancer. Approximately 75% of patients with low rectal cancer underwent a sphincter-preserving surgery; and the overall trend in sphincter-preserving surgery increased 3.6% per year over the last 20 years. Utilization of ISR/CSPO and laparoscopic surgery had the fastest annual increase since the application of the techniques at the hospital (49.4% and 15.1% annually, respectively) and in the past five years (32.4% and 33.1% annually, respectively). The probability of a sphincter preservation increased annually for ultra-low and lower tumors adjacent to the sphincter complex over the last 20 years. Moreover, patients with low rectal cancer who underwent sphincter preservation had better survival than those who underwent APR.

The overall proportion of sphincter-preserving surgeries for patients with low rectal cancer was 75%, ranging from 50% to 88% over the last 20 years in China. The sphincter-preserving proportion varies between countries and time periods (25). A national-wide study in England reported an overall proportion of sphincter-preserving surgery, presented as anterior resection procedure, was 75% for rectal cancer patients, ranging from 71.6% to 78.8% for the years 1996 to 2004 (31). However, the study was for rectal cancer and the sphincter-preserving proportion would be lower than 75% if the population was restricted to low rectal tumors. From a review of 2010-2015 National Cancer Database (NCDB) in the US, the sphincter-preserving proportion was 79% for rectal cancer (32) and researchers estimated that the rate was <60% for low rectal cancers (25). Although it was challenging to make an objective explanation for a varied sphincter-preserving proportions among countries, the high-volume and specialization might favor a higher rate for sphincter preservation (25).

Our study demonstrated that ISR/CSPO had the most rapid increase per year after initiation in 2013, while LAR was the most performed sphincter-preserving procedure with a steady trend over the last 20 years. AR was the first effective sphincter-preserving surgery developed by Dixon in 1948 (11), and was also the most performed sphincter-preserving procedure across different countries, with a performance rate of 60%-80% of all sphincter-preserving surgeries (31–34). Intersphincteric resection (ISR) was introduced by Schiessel et al. in 1994 (12), and was rapidly expanded to European and Asian countries in the 2000s (35, 36). However, ISR could not avoid the functional impairment of anal sphincter. At Changhai Hospital, ISR was modified by Zhang et al. in the 2010s to CSPO for functional keeping through preserving more dentate line and distal rectal wall (6, 37). Among 177 patients with ISR/CSPO, 160 (90.4%) patients underwent CSPO and 17 (9.6%) did ISR. Although the overall proportion of ISR/CSPO (5.6%) among sphincter-preserving surgeries observed in our study was lower than other centers (10-20%) (38), ISR/CSPO was a promising surgery regarding its acceptable oncological and functional outcomes in sphincter-preserving surgery (37).

We further observed that sphincter preservation increased annually for patients with ultra-low and very low tumors, probably driven by the development of surgical techniques and surgeons’ experience (39), along with the advance of neoadjuvant therapy (40, 41). In the present cohort, 720 patients who had ultra-low tumors ≤2cm from the anal verge would have required APR if treated traditionally. However, approximately one-third of those patients underwent a sphincter-preserving surgery at the hospital. The decision between a sphincter-preserving surgery and APR was more likely dependent on the infiltration of the external anal sphincter, than the conventional tumor distance from the anal verge (5). Patients with ultra-low rectal tumors but not invading the external sphincter would still have the opportunity for a sphincter-preserving surgery.

In our study, laparoscopic approach increased rapidly in the last five years and gradually became the predominant surgical technique. Laparoscopy was introduced in the 1980s for colorectal surgery and has been broadly accepted since 2000s (42). It is safe and feasible regarding recovery, physiological function, and short-term oncologic outcomes compared to open surgery (14, 15). The NCDB in the US during 2010-2015 reported about 40% of rectal cancer surgeries were laparoscopic (32). At Changhai Hospital, laparoscopic technique was first performed in 2009 for a sphincter-preserving surgery; almost half of the surgeries were laparoscopic in the last two years. However, the laparoscopic approach was still challenging for rectal cancers due to the straight rigid instruments and a narrow deep pelvic cavity, which were largely dependent on the experience of surgeons (39).

We observed that patients who had undergone sphincter-preserving surgery had a lower risk of death compared to APR, consistent with previous studies in US (33), France (25) and Korea (36). The French GRECCAR study reported a 10-year overall survival for sphincter-preserving surgery of 72.2%, higher than the APR surgery of 54.7% among ultra-low rectal tumors (25). A propensity score-matched SEER analysis also reported better 5-year overall survival for a sphincter-preserving surgery of 76.7% compared to APR of 65.6% (33). APR was revealed to be associated with a higher rate of perforation, local recurrence and positive margins than a sphincter-preserving surgery (23, 43). Although the extralevator APR had been developed to avoid wasting of the specimen and tumor perforation; tumor perforation might still happen at the anterior wall of rectum. Moreover, in our study, we observed a decreasing proportion of APR in the last 20 years, and the overall survival among low rectal cancer patients developed over time.

### Strengths and limitations

Our study is the first to quantify the trend of sphincter-preserving surgery in Chinese patients with low rectal cancer. The data covered a time period of more than 20 years and a sample size of over 4,000 patients with low rectal cancer, compared with other real-world studies with fewer than 250 patients (15, 44). The long-term time frame and a large sample size provided a fundamental source for trend analysis and estimation of survival status. Moreover, our data were mainly from EMRs, which provided detailed information on both inpatient and outpatient health care based on clinicians’ routine practice.

This study had several limitations. First, our data came from a single center for investigation. The patients included in our study might not be representative of all patients with low rectal cancer in China; and surgical development had a large variation for different hospitals in China. Additionally, follow-up information from the outpatient records at Changhai Hospital was missing for some patients. Patients who were not residents of Shanghai might have been followed up at their local hospitals. Follow-up through phone interviews might have recall bias. However, the characteristics of patients followed-up and non-follow-up were comparable (**Supplemental Table 1**), and sensitivity analyses for multiple follow-up intervals had consistent results. Furthermore, the joinpoint method was a bivariate trend method which did not adjust for potential changes in patient characteristics over time. Therefore, the observed trends might be partially reflective of such concurrent changes over time, if any were present.

## Conclusions

Utilization of sphincter-preserving surgeries for low rectal cancer increased significantly over the last 20 years in a real-world setting in China. The overall trend in sphincter-preserving surgery for low-lying tumors increased 3.6% per year from 1999-2021. Utilization of ISR/CSPO and laparoscopic surgery had the fastest annual increase since the application of the techniques at the hospital. Patients with low lying rectal cancer who underwent sphincter preservation had better survival than APR. Future multi-center studies from various geographic areas in China are needed for trend analysis. With improved survival for low rectal cancer, evidence regarding patients’ quality of life benefited from the novel surgical technique along with the multidisciplinary treatment is also needed and may contribute to a better understanding of sphincter function and intestinal continuity after sphincter preservation.

## Data availability statement

The dataset is for clinical routine practice and is available on reasonable request from the corresponding author WZ at Changhai Hospital, the First Affiliated Hospital of Naval Medical University, Shanghai, China. Requests to access these datasets should be directed to WZ; weizhang2000cn@163.com.

## Author contributions

KZ, QH and GY contributed to the study design, data collection, statistical analysis and manuscript drafting. YoZ, YY and YuZ contributed to the data curation and visualization. HW, LH, ZL, LZ and EY contributed to the data interpretation and cirtical revision. HQ, RM and WZ contributed to the study design, supervision and critical revision. All authors reviewed and approved the final manuscript.

## References

[1] Cao W Chen HD Yu YW Li N Chen WQ. Changing profiles of cancer burden worldwide and in China: A secondary analysis of the global cancer statistics 2020. Chin Med J (2021) 134(7):783–91. doi: 10.1097/CM9.0000000000001474 PMC8104205 33734139

[2] Zhu J Tan Z Hollis-Hansen K Zhang Y Yu C Li Y. Epidemiological trends in colorectal cancer in China: An ecological study. Digest Dis Sci (2017) 62(1):235–43. doi: 10.1007/s10620-016-4362-4 27796769

[3] Wang J Liu L Cai Y Gao Y Guo Z Yu F. Trends in the age-related incidence of colon and rectal cancers in China, 2005-2015. Dig Liver Dis (2021) 53(7):908–14. doi: 10.1016/j.dld.2021.01.009 33551354

[4] Ferlay J Laversanne M Ervik M Lam F Colombet M Mery L. Global cancer observatory: Cancer tomorrow (2020). Lyon, France: International Agency for Research on Cancer. Available at: https://gco.iarc.fr/tomorrow (Accessed June 01 2021).

[5] Rullier E Laurent C Bretagnol F Rullier A Vendrely V Zerbib F. Sphincter-saving resection for all rectal carcinomas: The end of the 2-cm distal rule. Ann Surg (2005) 241(3):465–9. doi: 10.1097/01.sla.0000154551.06768.e1 PMC1356985 15729069

[6] Sun G Lou Z Zhang H Yu GY Zheng K Gao XH. Retrospective study of the functional and oncological outcomes of conformal sphincter preservation operation in the treatment of very low rectal cancer. Tech Coloproctol (2020) 24(10):1025–34. doi: 10.1007/s10151-020-02229-2 PMC7522072 32361871

[7] Haward RA Morris E Monson JR Johnston C Forman D. The long term survival of rectal cancer patients following abdominoperineal and anterior resection: Results of a population-based observational study. Eur J Surg Oncol (2005) 31(1):22–8. doi: 10.1016/j.ejso.2004.08.002 15642422

[8] Rullier E Denost Q Vendrely V Rullier A Laurent C. Low rectal cancer: Classification and standardization of surgery. Dis Colon Rectum (2013) 56(5):560–7. doi: 10.1097/DCR.0b013e31827c4a8c 23575394

[9] Nicholls R Hall C. Treatment of non-disseminated cancer of the lower rectum. J Br Surg (1996) 83(1):15–8. doi: 10.1002/bjs.1800830105 8653351

[10] Maslyankov S Penchev D Todorov G Vladov N. A meta-analysis of quality of life, estimated by questionnaires of the European organization for research and treatment of cancer (EORTC) after rectal cancer surgery. Chirurgia (2015) 110(4):356–61. 26305200

[11] Dixon CF. Anterior resection for malignant lesions of the upper part of the rectum and lower part of the sigmoid. Ann Surg (1948) 128(3):425–42. doi: 10.1097/00000658-194809000-00009 PMC1514072 17859211

[12] Schiessel R Karner-Hanusch J Herbst F Teleky B Wunderlich M. Intersphincteric resection for low rectal tumours. Br J Surg (1994) 81(9):1376–8. doi: 10.1002/bjs.1800810944 7953423

[13] Tilney H Tekkis P. Extending the horizons of restorative rectal surgery: Intersphincteric resection for low rectal cancer. Colorectal Dis (2008) 10(1):3–15. doi: 10.1111/.1463-1318.2007.01226.x 17477848

[14] Kuper MA Eisner F Konigsrainer A Glatzle J. Laparoscopic surgery for benign and malign diseases of the digestive system: Indications, limitations, and evidence. World J Gastroenterol (2014) 20(17):4883–91. doi: 10.3748/wjg.v20.i17.4883 PMC4009519 24803799

[15] Zhang X Wu Q Hu T Gu C Bi L Wang Z. Laparoscopic versus conventional open surgery in intersphincteric resection for low rectal cancer: A systematic review and meta-analysis. J Laparoendosc Adv Surg Tech A (2018) 28(2):189–200. doi: 10.1089/lap.2017.0495 29232537

[16] Huang YM Huang YJ Wei PL. Outcomes of robotic versus laparoscopic surgery for mid and low rectal cancer after neoadjuvant chemoradiation therapy and the effect of learning curve. Med (Baltimore) (2017) 96(40):e8171. doi: 10.1097/MD.0000000000008171 PMC5738003 28984767

[17] Han JG Wei GH Gao ZG Zheng Y Wang ZJ. Intersphincteric resection with direct coloanal anastomosis for ultralow rectal cancer: The experience of people’s republic of China. Dis Colon Rectum (2009) 52(5):950–7. doi: 10.1007/DCR.0b013e31819f13a3 19502861

[18] Jeong S-Y Park JW Nam BH Kim S Kang S-B Lim S-B. Open versus laparoscopic surgery for mid-rectal or low-rectal cancer after neoadjuvant chemoradiotherapy (COREAN trial): Survival outcomes of an open-label, non-inferiority, randomised controlled trial. Lancet Oncol (2014) 15(7):767–74. doi: 10.1016/S1470-2045(14)70205-0 24837215

[19] Li SY Chen G Bai X Zuo FY Chen G Du JF. Anus-preserving rectectomy *via* telescopic colorectal mucosal anastomosis for low rectal cancer: Experience from a Chinese cohort. World J Gastroenterol (2013) 19(24):3841–6. doi: 10.3748/wjg.v19.i24.3841 PMC3699045 23840123

[20] Nagtegaal ID van de Velde CJ Marijnen CA van Krieken JH Quirke P Dutch Colorectal Cancer G. Low rectal cancer: A call for a change of approach in abdominoperineal resection. J Clin Oncol (2005) 23(36):9257–64. doi: 10.1200/JCO.2005.02.9231 16361623

[21] Park EJ Cho MS Baek SJ Hur H Min BS Baik SH. Long-term oncologic outcomes of robotic low anterior resection for rectal cancer: A comparative study with laparoscopic surgery. Ann Surg (2015) 261(1):129–37. doi: 10.1097/SLA.0000000000000613 24662411

[22] Park JS Choi GS Lim KH Jang YS Jun SH. Robotic-assisted versus laparoscopic surgery for low rectal cancer: Case-matched analysis of short-term outcomes. Ann Surg Oncol (2010) 17(12):3195–202. doi: 10.1245/s10434-010-1162-5 20589436

[23] Piozzi GN Baek SJ Kwak JM Kim J Kim SH. Anus-preserving surgery in advanced low-lying rectal cancer: A perspective on oncological safety of intersphincteric resection. Cancers (2021) 13(19):4793. doi: 10.3390/cancers13194793 PMC8507715 34638278

[24] Rouanet P Rivoire M Gourgou S Lelong B Rullier E Jafari M. Sphincter-saving surgery after neoadjuvant therapy for ultra-low rectal cancer where abdominoperineal resection was indicated: 10-year results of the GRECCAR 1 trial. Br J Surg (2021) 108(1):10–3. doi: 10.1093/bjs/znaa010 33640922

[25] Rouanet P Rivoire M Gourgou S Lelong B Rullier E Jafari M. Sphincter-saving surgery for ultra-low rectal carcinoma initially indicated for abdominoperineal resection: Is it safe on a long-term follow-up? J Surg Oncol (2021) 123(1):299–310. doi: 10.1002/jso.26249 33098678

[26] Joinpoint Trend Analysis Software. (2022). Available at: https://surveillance.cancer.gov/joinpoint/ (Accessed April 13, 2022).

[27] VanderWeele TJ. Principles of confounder selection. Eur J Epidemiol (2019) 34(3):211–9. doi: 10.1007/s10654-019-00494-6 PMC6447501 30840181

[28] Jager KJ Zoccali C Macleod A Dekker FW. Confounding: What it is and how to deal with it. Kidney Int (2008) 73(3):256–60. doi: 10.1038/sj.ki.5002650 17978811

[29] Grambsch PM Therneau TM. Proportional hazards tests and diagnostics based on weighted residuals. Biometrika (1994) 81(3):515–26. doi: 10.1093/biomet/81.3.515

[30] RStudio. RStudio: Integrated development for r. Boston: RStudio Team (2020).

[31] Tilney HS Heriot AG Purkayastha S Antoniou A Aylin P Darzi AW. A national perspective on the decline of abdominoperineal resection for rectal cancer. Ann Surg (2008) 247(1):77–84. doi: 10.1097/SLA.0b013e31816076c3 18156926

[32] Keller DS Reif de Paula T Kiran RP. Ready for the national accreditation programs for rectal cancer? auditing rectal cancer outcomes in the united states. Colorectal Dis (2019) 21(10):1213–5. doi: 10.1111/codi.14729 31206230

[33] Warschkow R Ebinger SM Brunner W Schmied BM Marti L. Survival after abdominoperineal and sphincter-preserving resection in nonmetastatic rectal cancer: A population-based time-trend and propensity score-matched SEER analysis. Gastroenterol Res Pract (2017) 2017:6058907. doi: 10.1155/2017/6058907 28197206 PMC5286526

[34] Marwan K Staples MP Thursfield V Bell SW. The rate of abdominoperineal resections for rectal cancer in the state of Victoria, Australia: A population-based study. Dis Colon Rectum (2010) 53(12):1645–51. doi: 10.1007/DCR.0b013e3181f46485 21178859

[35] Rullier E Zerbib F Laurent C Bonnel C Caudry M Saric J. Intersphincteric resection with excision of internal anal sphincter for conservative treatment of very low rectal cancer. Dis Colon Rectum (1999) 42(9):1168–75. doi: 10.1007/BF02238569 10496557

[36] Kim HS Ko S Oh NG. Long-term results of extended intersphincteric resection for very low rectal cancer: A retrospective study. BMC Surg (2016) 16:21. doi: 10.1186/s12893-016-0133-6 27090553 PMC4835892

[37] Zhang W Lou Z. Conformal sphincter-preserving operation + NOSES I (CSPO + NOSES I) for extremely low rectal cancer. In: Wang X, editor. Natural orifice specimen extraction surgery: Gastrointestinal tumor. Singapore: Springer Singapore (2021). p. 663–6.

[38] Ogura A Konishi T Cunningham C Garcia-Aguilar J Iversen H Toda S. Neoadjuvant (chemo) radiotherapy with total mesorectal excision only is not sufficient to prevent lateral local recurrence in enlarged nodes: Results of the multicenter lateral node study of patients with low cT3/4 rectal cancer consortium. J Clin Oncol (2019) 37(1):33–43. doi: 10.1200/JCO.18.00032 30403572 PMC6366816

[39] Guillou PJ Quirke P Thorpe H Walker J Jayne DG Smith AMH. Short-term endpoints of conventional versus laparoscopic-assisted surgery in patients with colorectal cancer (MRC CLASICC trial): Multicentre, randomised controlled trial. Lancet (2005) 365(9472):1718–26. doi: 10.1016/S0140-6736(05)66545-2 15894098

[40] Kinoshita H Watanabe T Yanagisawa A Nagawa H Kato Y Muto T. Pathological changes of advanced lower-rectal cancer by preoperative radiotherapy. Hepatogastroenterology (2004) 51(59):1362–6. 15362753

[41] Lefevre JH Mineur L Kotti S Rullier E Rouanet P de Chaisemartin C. Effect of interval (7 or 11 weeks) between neoadjuvant radiochemotherapy and surgery on complete pathologic response in rectal cancer: A multicenter, randomized, controlled trial (GRECCAR-6). J Clin Oncol (2016) 34(31):3773–80. doi: 10.1200/JCO.2016.67.6049 27432930

[42] Semm K. Endoscopic appendectomy. Endoscopy (1983) 15(2):59–64. doi: 10.1055/s-2007-1021466 6221925

[43] Wang XT Li DG Li L Kong FB Pang LM Mai W. Meta-analysis of oncological outcome after abdominoperineal resection or low anterior resection for lower rectal cancer. Pathol Oncol Res (2015) 21(1):19–27. doi: 10.1007/s12253-014-9863-x 25430561 PMC4287681

[44] Shirouzu K Murakami N Akagi Y. Intersphincteric resection for very low rectal cancer: A review of the updated literature. Ann Gastroenterol Surg (2017) 1(1):24–32. doi: 10.1002/ags3.12003 29863144 PMC5881339

