# Supplementary material for: Trends of sphincter-preserving surgeries for low lying rectal cancer: A 20-year experience in China
Source: Front Oncol. 2022 Dec 8;12:996866. doi: 10.3389/fonc.2022.996866 (PMC9773833; doi:10.3389/fonc.2022.996866)
Supplement: Supplementary file 1 [file DataSheet_1.docx]

Supplemental figure 1. The flowchart of the study population


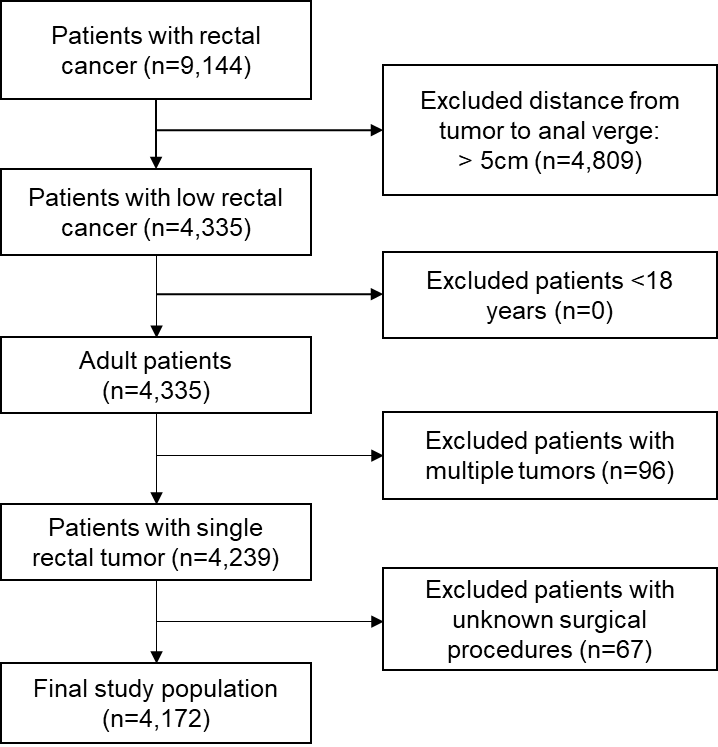


Supplemental table 1. The characteristics of patients with non-follow-up and follow-up in our cohort.

|  | Non-follow-up (n=1,242) | Follow-up (n=2,930) |
| --- | --- | --- |
| Age, years, mean (SD) | 58.2 (12.4) | 59.8 (11.6) |
| Gender |  |  |
| Female | 467 (40.0) | 1099 (37.5) |
| Male | 775 (60.0) | 1831 (62.5) |
| Comorbidities | 596 (48.0) | 1509 (51.5) |
| Tumor location, cm, median (IQR)^1^ | 4 (3.0-5.0) | 4 (3.0-5.0) |
| Tumor location group |  |  |
| ≤2cm | 225 (18.1) | 475 (16.2) |
| 2-≤3cm | 304 (24.5) | 724 (24.7) |
| 3-≤4cm | 330 (26.6) | 855 (29.2) |
| 4-≤5cm | 383 (30.8) | 876 (29.9) |
| Surgical procedure |  |  |
| Hartmann | 20 (1.6) | 47 (1.6) |
| LAR/AR | 772 (62.2) | 1954 (66.7) |
| Trans-anal local excision | 67 (5.4) | 129 (4.4) |
| ISR/CSPO | 75 (6.0) | 179 (6.1) |
| APR | 308 (24.8) | 624 (21.2) |
| Surgical approach |  |  |
| Open | 965 (77.7) | 2,209 (75.4) |
| Laparoscopy | 240 (19.3) | 592 (20.2) |
| Other ^2^ | 37 (3.0) | 129 (4.4) |
| Neoadjuvant therapy | 191 (15.4) | 571 (19.5) |
| Hospitalization days, median (IQR) | 11 (8-15) | 7 (5-9) |
| Pathological stage |  |  |
| Ⅰ | 351 (28.30) | 823 (28.10) |
| Ⅱ | 312 (25.10) | 753 (25.70) |
| Ⅲ | 451 (36.30) | 1,055 (36.60) |
| Others | 128 (10.30) | 270 (9.20) |
| Histological grading^3^ |  |  |
| Grade1 | 27 (2.20) | 47 (1.60) |
| Grade2 | 944 (76.00) | 2279 (77.80) |
| Grade3 | 107 (8.50) | 287 (9.80) |
| Missing | 164 (13.20) | 305 (10.40) |
| Histological classification |  |  |
| Common type of adenocarcinoma | 995 (80.10) | 2391 (81.60) |
| Special type of adenocarcinoma | 138 (11.10) | 316 (10.80) |
| Other ^4^ | 109 (8.80) | 1. 7.40) |

Abbreviations: APR: abdominoperineal resection; AR: anterior resection; LAR: low anterior resection; ISR: intersphincteric resection; CSPO: contoured resection of rectum

1. Tumor location was measured through colonoscopy or rigid sigmoidoscope / proctoscopy in clinical examination before surgery.
2. Trans-anal resections
3. Included non-epithelial tumors (myogenic tumors, neurogenic tumors, GIST (gastrointestinal stromal tumors), lipomas and lipomatosis, tumor blood vessels, and other tumors).
4. Grade 1 - well differentiated; Grade 2 - moderately differentiated; Grade 3 - Poorly Differentiated; Grade 4 – Undifferentiated.

Supplemental table 2. Trends analyses for specific groups in sphincter-preserving percentages between 2000-2021.

| Specific groups | | AAPC | 95% CI |
| --- | --- | --- | --- |
|  | Year of surgery |  |  |
|  | 2000-2008 | 5.8^*^ | 2.7-9.1 |
|  | 2009-2015 | 1.4 | -0.4-3.3 |
|  | 2016-2021 | -0.7 | -4.1-2.8 |
|  | Age group, years |  |  |
|  | 18-45 | 1.8 | 0.6-3.0 |
|  | 46-65 | 3.1^*^ | 2.1-4.1 |
|  | 66-97 | 3.1^*^ | 2.2-4.1 |
|  | Gender |  |  |
|  | Female | 2.0 | 0.7-3.2 |
|  | Male | 4.6^*^ | 2.8-6.4 |

Abbreviation: AAPC: average annual percentage change

∗ The APC is significantly different from zero (p < 0.05).

Supplemental table 3. Hazard ratios (HRs) for overall mortality among patients undergone sphincter-preserving surgery compared with APR by followed up periods.

| Sphincter-preserving surgery (vs. APR) | HR ^a^ (95% CI) | P |
| --- | --- | --- |
| Follow-up periods |  |  |
| One year | 0.88 (0.67-1.16) | 0.37 |
| Three years | 0.74 (0.60-0.93) | 0.01 |
| Five years | 0.75 (0.61-0.93) | 0.01 |

Abbreviations. APR, Abdominoperineal resection.

1. Models were adjusted for demographic, clinical, pathological and treatment characteristics, including year of surgery groups, age groups, gender, tumor location groups, tumor size, baseline comorbidities, neoadjuvant therapy, pathological stage, histology, grading.
